# A Bibliometric Analysis of Accelerated Orthodontics Research in the Middle East Across Three Major Databases

**DOI:** 10.7759/cureus.109792

**Published:** 2026-05-28

**Authors:** Obadah Hassan Alkaddeh, Mohammad Y. Hajeer, Huda Abutayyem, Mohammed A. Awawdeh, Alaa Oudah Ali Almusawi, Ahmad S. Zakaria

**Affiliations:** 1 Department of Orthodontics, Faculty of Dentistry, Damascus University, Damascus, SYR; 2 Department of Clinical Sciences, Center of Medical and Bio-Allied Health Sciences Research, College of Dentistry, Ajman University, Ajman, ARE; 3 Department of Preventive Dental Science, College of Dentistry, King Saud bin Abdulaziz University for Health Sciences, Riyadh, SAU; 4 Department of Orthodontics, Faculty of Dentistry, Al-Kunooze University College, Basrah, IRQ; 5 Department of Orthodontics, School of Dental Sciences, Universiti Sains Malaysia, Kota Bharu, MYS

**Keywords:** accelerated orthodontics, canine retraction, corticotomy, electric simulation, en masse retraction, low-level laser therapy (lllt), micro-osteoperforation (mops), middle east, orthodontic tooth movement, piezocision

## Abstract

Accelerated orthodontics seeks to reduce the prolonged duration of conventional orthodontic treatment and the associated pain and discomfort, particularly among adult patients. A growing body of invasive, minimally invasive, and noninvasive techniques, ranging from corticotomy and periodontally accelerated osteogenic orthodontics to piezocision, micro-osteoperforations, and low-level laser therapy (LLLT), has stimulated regional research activity and prompted the need to map scholarly output and trends across the Middle East. The objective of this work was to perform a comprehensive bibliometric analysis of accelerated orthodontics publications originating from Middle Eastern countries (2008-2025), identifying publication trends, leading authors and institutions, dominant research themes, citation impact, and patterns of regional collaboration to inform future clinical research priorities. A systematic search of PubMed, Scopus, and Web of Science was conducted in September 2025 using a predefined strategy. The PICOS criteria included adult patients (≥18 years) receiving fixed or removable orthodontic treatment with adjunctive acceleration methods; comparators were conventional treatment without acceleration; outcomes were treatment duration or rate of tooth movement; and eligible study designs were randomized controlled trials, cohort studies, and systematic reviews published in English between 2005 and 2025. Search results from the three databases were exported to EndNote and analyzed with the VOSviewer software (version 1.6.20) to generate co-authorship, co-citation, and keyword networks. Two independent reviewers screened titles/abstracts and full texts, with a third reviewer resolving discrepancies. Extracted bibliometric indicators included publication counts, citation totals, author and institutional affiliations, article types, and keyword clusters. Following the predefined search strategy, a total of 973 articles were initially identified and screened for relevance. After this screening process, 65 publications were included in the final analysis. The final corpus comprised 48 randomized controlled trials (76%), 16 systematic reviews (23%), and one cohort study (1%). Annual output and citations rose markedly from 2013 onward, with an exponential surge between 2021 and 2024 and a peak in 2022 (19 articles; 320 citations). A total of 120 authors contributed; the most prolific authors were Hajeer MY, Burhan AS, and Ajaj MA, with 14 of the top 15 authors affiliated with Syria. Damascus University produced 52 publications, and Syria led citation impact (1,772 citations), followed by Turkey and Saudi Arabia. Keyword analysis revealed four thematic clusters: pain and discomfort, acceleration methods (surgical, minimally invasive, and noninvasive), target populations, and gender. The most cited works focused on surgical approaches and LLLT. Accelerated orthodontics research from the Middle East has grown substantially, driven predominantly by Syrian institutions, especially Damascus University, and concentrated author networks. Surgical techniques and LLLT dominate high-impact publications, while minimally invasive and noninvasive modalities are increasingly investigated and appear more acceptable to certain patient groups. Broader multicenter and international trials, standardized outcome measures, and patient-centered evaluations are recommended to translate regional advances into generalizable clinical guidelines.

## Introduction and background

The aim of orthodontic treatment is to achieve perfect alignment of teeth and maximum intercuspation, which requires a period of time ranging between 24 and 36 months, which is considered a relatively long time [1]. The length of the treatment period is one of the most important problems facing orthodontic patients, in addition to the pain and discomfort associated with the treatment [2]. Most of the concerns about the duration of treatment time, pain, and discomfort associated with wearing orthodontic appliances are often associated with adult patients [3]. Recently, the field of accelerated orthodontics has gained extensive interest from researchers and healthcare professionals who are looking for new ways to improve orthodontic treatment [4].

Many treatment methods have been developed to accelerate orthodontic tooth movement (OTM), including invasive, minimally invasive, and noninvasive techniques [5]. Invasive methods, such as periodontally accelerated osteogenic orthodontics (PAOO) [6], corticotomy [7], and interseptal alveolar surgery [8], have strong evidence for reducing orthodontic treatment time but are not common in daily practice due to their aggressive nature and their potential complications [9].

Consequently, invasive and minimally invasive techniques have been used in orthodontics in the past decade, such as corticision [10], piezocision [9], cortico-alveolar perforations [11], corticotomy [12], and laser-assisted flapless corticotomy [13]. Conversely, noninvasive methods, such as low-level laser therapy (LLLT) [14], vibration therapy [15], light-emitting diodes [16], pulsed electromagnetic waves [17], and magnetotherapy [18], are being widely researched due to their safety and easy implementation. Additionally, the use of self-ligating brackets is considered one of the methods to accelerate OTM, which is being used due to its promising results [19]. Another method of accelerating orthodontic treatment is taking advantage of the frictional and the nonfrictional methods in orthodontic practice to reduce treatment time [20]. Furthermore, the administration of drugs and biological products, such as the injection of platelet-rich plasma and platelet-rich fibrinogen, has been studied and investigated [21].

In the past years, there has been a rising number of studies and reviews in the field of accelerated OTM, indicating the importance of this topic in orthodontics worldwide. A bibliometric analysis of international articles studying accelerated orthodontics has shown an aggregation of these articles issued from the Middle East countries, which emphasizes their interest in this topic and their impact on the development of accelerated orthodontics [22]. The large number of studies from the Middle East covering different methods of acceleration made a significant impact in various bibliometric analyses interested in accelerated orthodontics [4].

However, even though there have been several bibliometric analyses of articles studying various methods of acceleration, there has been no quantitative, statistical, and computational analysis of the published articles from the Middle East. Consequently, there is a pressing need to conduct a comprehensive quantitative bibliometric analysis focused on accelerated orthodontics within the Middle East. This analysis aims to provide a comprehensive understanding of the current state of accelerated orthodontic research in the Middle East by systematically evaluating the published articles. Its primary objectives are to uncover key scientific profiles, emerging trends, and critical parameters, including authorship patterns, the geographic distribution of contributing institutions, influential authors, countries/regions, and journals. Through keyword and citation analysis, the study seeks to identify key contributors and illustrate the current research status and topics. A central aim is to evaluate publication trends in the past decade and across diverse geographic regions within the Middle East to guide clinical advancements in the field of accelerated orthodontics.

## Review

Materials and methods

Data Sources and Search Strategy

A comprehensive literature search was performed in the PubMed, Scopus, and Web of Science databases in September 2025 and was carefully designed to include all relevant studies on accelerated orthodontics, ensuring thorough and precise data collection. The detailed search strategy is included in Appendix A.

The PICOS framework was set as follows: Participants: Healthy male and female patients aged 18 years or older with any type of malocclusion and from any ethnic group who received treatment with fixed or removable orthodontic appliances. Intervention: Fixed orthodontic treatment associated with any accelerating method as an adjunctive procedure. Comparison: Fixed orthodontic treatment without any accelerating interventions. Outcome measures: Treatment duration, rate of OTM, or treatment time reduction were the primary outcomes. Study design: randomized controlled trials (RCTs), cohort, and systematic review studies published between 2005 and 2025, in the English language only.

The selection process consisted of two stages. First, titles and abstracts were screened to eliminate nonrelevant publications. Then, the full texts of the remaining articles were retrieved and thoroughly examined to ensure they met the predefined inclusion criteria.

The resulting dataset was analyzed to extract key bibliometric indicators, including publication counts, citation counts, and the distribution of studies. The search results were exported from three databases into EndNote to collect all relevant articles, and the bundle was then exported to the VOSviewer software (version 1.6.20; Centre for Science and Technology Studies, Leiden University, Leiden, Netherlands) for further analysis.

Data Screening

To ensure the precision and dependability of the data collection process, two independent reviewers (OHA and HA) conducted rigorous cross-verification of the entered data, and a third reviewer (ASZ) was consulted to adjudicate any discrepancies identified during data screening. After the screening process, the reviewers compared their results and agreed on the publications to be included in the analysis. They meticulously examined various data elements, including titles, keywords, publication dates, authors, affiliated institutions, source journals, citation counts, and authors' national or regional affiliations. This stringent validation procedure effectively reduced errors and inconsistencies, thereby preserving the dataset's integrity for subsequent bibliometric analysis.

Data Extraction

General bibliometric indicators were extracted by two independent researchers (OHA and HA), with a third researcher (ASZ) consulted to resolve any disagreements from the selected publications, encompassing key elements such as titles, authors, publication years, citation counts, countries/regions, affiliated institutions, source journals, highly cited articles, references, and keywords to identify predominant research themes. The classification of article types was conducted through manual screening of titles, abstracts, and, where necessary, full texts.

Data Analysis and Findings Synthesis

A bibliometric analysis was conducted to examine the field of accelerated orthodontics in the Middle East from 2008 to 2025. Utilizing the VOSviewer software, key indicators, including publication titles, authors, years, citations, affiliations, journals, references, and keywords, were extracted from the selected literature. This enabled a comprehensive evaluation of annual scientific output, citation impact, prominent sources and institutions, keyword trends, leading authors, and collaborative patterns. Networks specific to accelerated OTM in the Middle East were also generated using VOSviewer.

Results

Retrieved Studies

**Figure 1:**
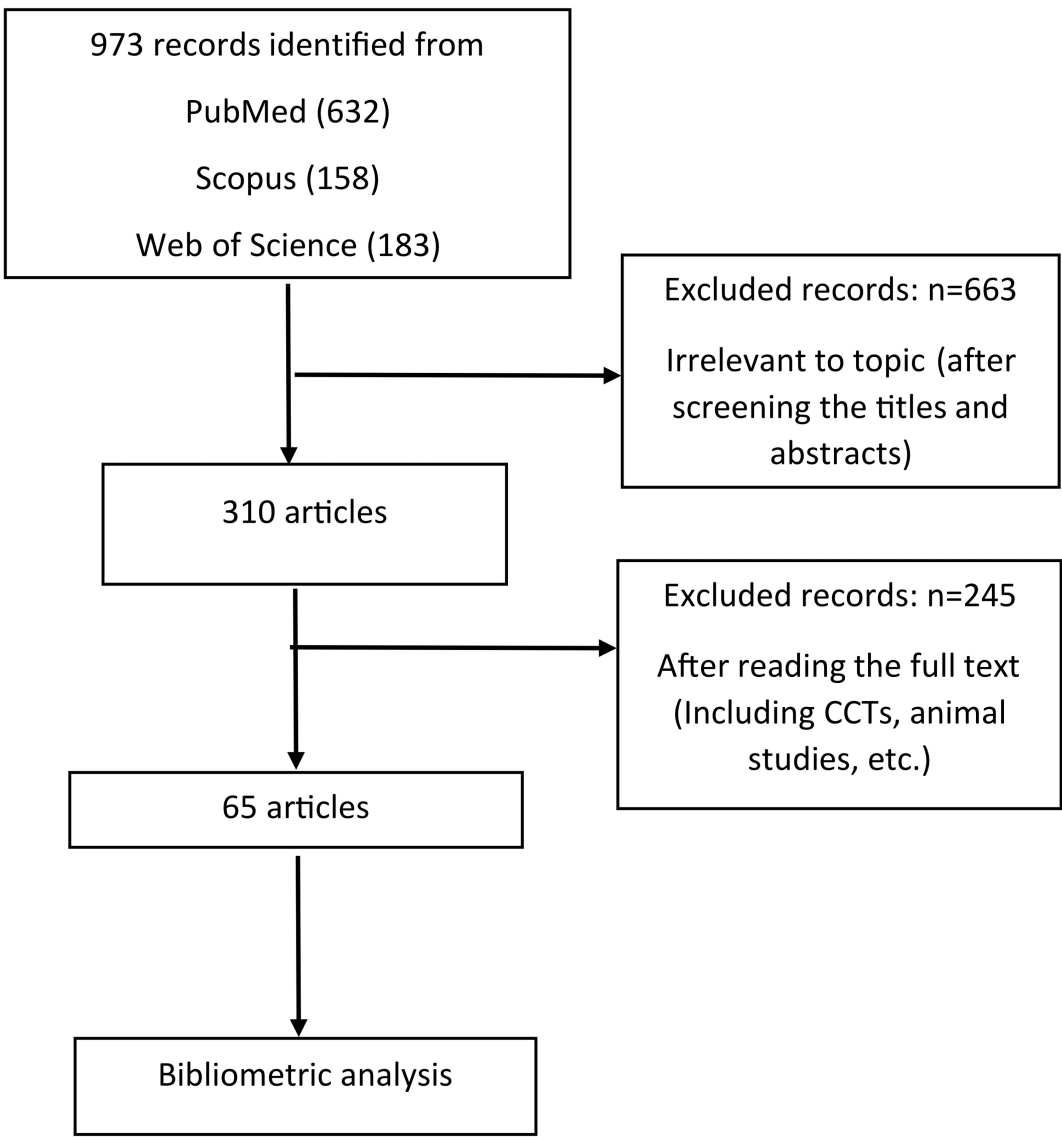
Flow chart of the data extraction process CCT, controlled clinical trial

The process began with the identification of 973 records from three major databases (PubMed, Scopus, and Web of Science). The initial screening phase excluded 663 records as irrelevant based on their titles and abstracts, yielding 310 articles for full-text review. A subsequent eligibility assessment excluded an additional 245 records, primarily because they did not meet the inclusion criteria. The final corpus for the bibliometric analysis consisted of 65 articles that successfully passed all stages of this rigorous screening protocol (Figure 1). According to the search strategy, a total of 65 articles, divided into 16 systematic reviews (23%), 48 RCTs (76%), and one cohort study (1%), were included in the analysis.

Publications Trends

**Figure 2:**
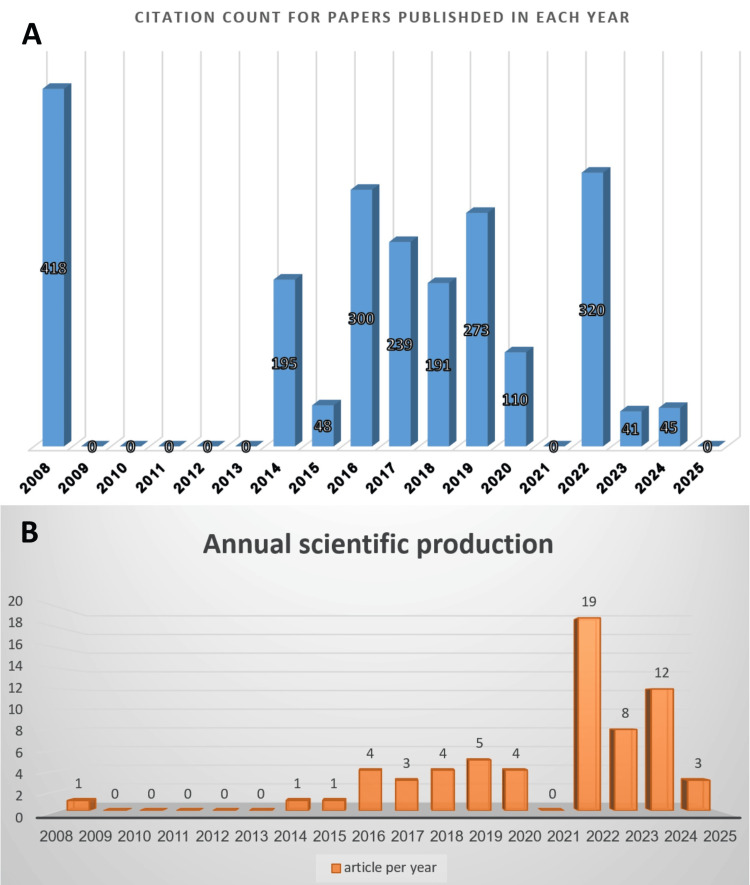
Timeline for included articles (A) Bar chart for the sum of citations for the included studies grouped by their year of publication. (B) Bar chart of annual scientific production.

**Figure 3:**
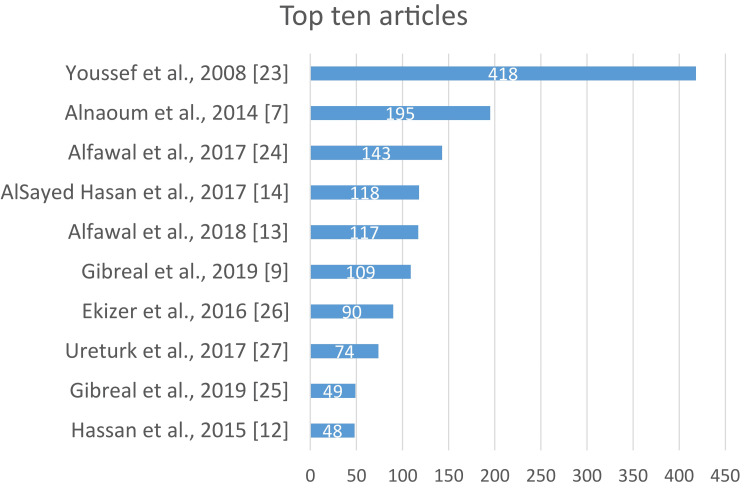
List of top 10 articles based on their citation count [7,9,12-14,23,24-27]

The analysis of annual citation data from 2008 to 2025 reveals a pronounced and sustained upward trajectory in scholarly impact, characterized by an initial period of minimal productivity from 2008 to 2012 [1,23]. This period was notably punctuated by a significant early contribution, as evidenced by a single article from 2008 receiving 418 citations [23], followed by a phase of consistent and accelerating growth from 2013 to 2020. This progression culminated in a period of exponential growth from 2021 to 2024, during which article numbers rose dramatically, reaching their peak in 2022 at 19 articles and 320 citations, thereby signaling a phase of peak influence and engagement within the academic community of the Middle East. The apparent decline in 2025 is attributed to incomplete data collection for the current year rather than a genuine reduction in scholarly attention (Figure 2A, 2B). A list of the top 10 articles based on their citation count is shown in Figure 3. Youssef et al. (2008) [23] is the most influential by far, with 418 citations, followed by Al-Naoum et al. (2014) [7] with 195 and Alfawal et al. (2016) [24] with 143. The remaining articles range from 118 to 48 citations, including two separate entries for Gibreal et al. (2019) [9,25] at 109 and 49 citations, respectively. Further down the ranking, Ekizer et al. (2016) [26] have received 90 citations, Üretürk et al. (2017) [27] have 74 citations, and Hassan et al. (2015) [12] round out the top 10 with 48 citations.

Author Analysis

**Figure 4:**
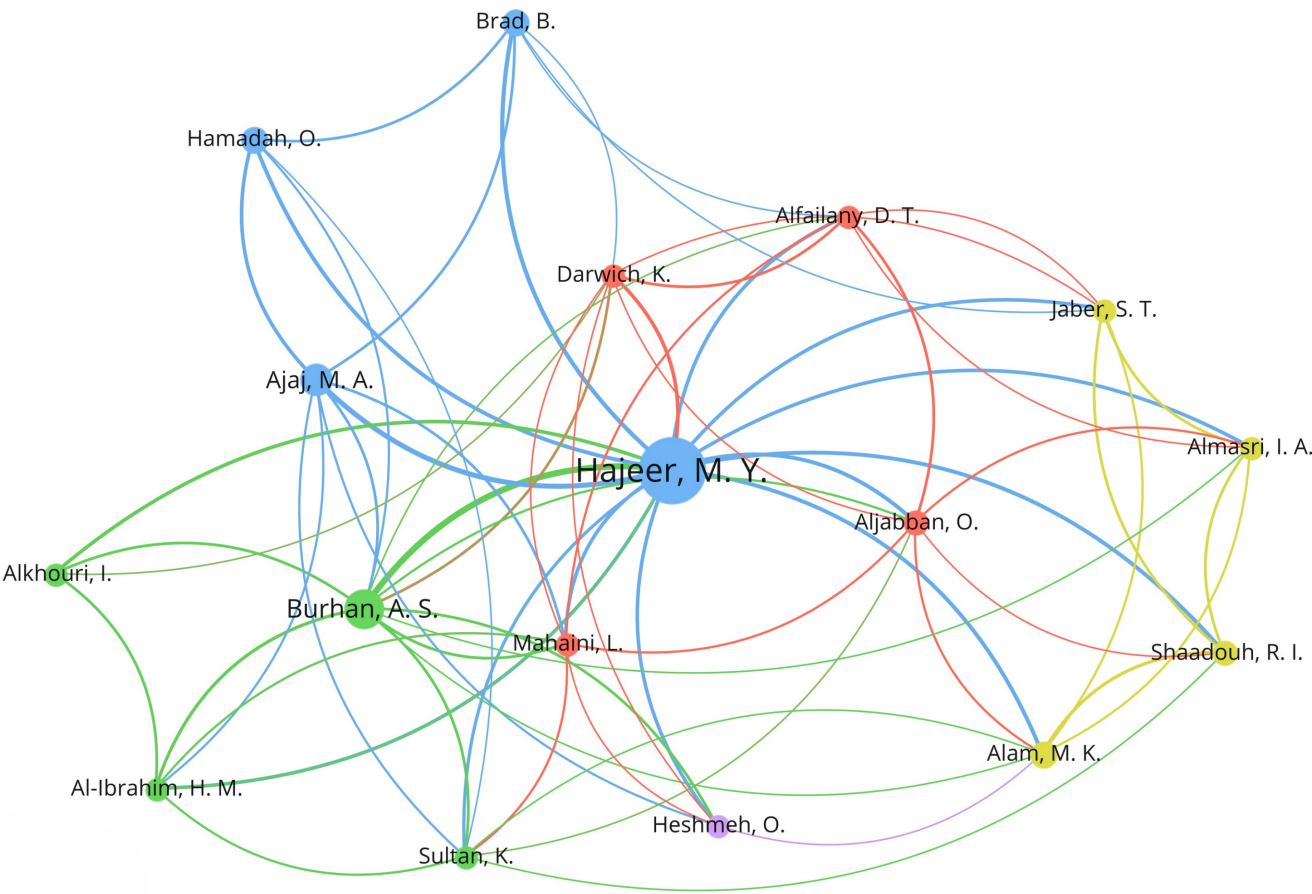
Visual network of authors’ collaborations This figure was created using VOSviewer software (version 1.6.20; Centre for Science and Technology Studies, Leiden University, Leiden, Netherlands).

Of the 65 articles of literature in this domain, a total of 120 authors were credited. The top three contributors are Hajeer MY (47 papers), Burhan AS (17 papers), and Ajaj MA (10 papers). A notable finding is that 14 of the top 15 authors are affiliated with Syria (Table 1), underscoring the country’s substantial influence in this field of orthodontic research. Identifying 17 influential authors with at least five articles in the field of accelerated orthodontics. The largest nodes correspond to the most frequently co-cited authors. The collaborative relationships among co-cited authors are visualized in Figure 4. The co-citation network demonstrates extensive connectivity among citations, reflecting relatively close relationships among the referenced studies.

**Table 1:** List of the top 15 authors along with the number of citations and country of origin

Author	Number of articles	Country of origin
Hajeer MY	47	Syria
Burhan AS	17	Syria
Ajaj MA	10	Syria
Alam MK	7	Saudi Arabia
Hamada O	7	Syria
Brad B	7	Syria
Aljabban O	6	Syria
Shaadouh RI	6	Syria
Mahaini I	5	Syria
Al-Ibrahim HM	5	Syria
Alfailany DT	5	Syria
Almasri IA	5	Syria
Darwich K	5	Syria
Sultan K	5	Syria
Jaber ST	5	Syria

Analysis of Countries and Regions

The most relevant affiliations contributing to research on accelerated orthodontics from 2008 to 2025 were identified, highlighting research institutions that have made significant contributions to this field. Topping the list is Damascus University, Syria, with 52 publications, followed by four different universities from Saudi Arabia with six publications and three different universities from Turkey with three publications (Table 2). Regarding citation impact, Syria had the highest cumulative citation count of 1772, followed by Turkey (170) and Saudi Arabia (115). These affiliations underscore their substantial role in advancing research in accelerated orthodontic methods.

**Table 2:** List of the top 10 universities in the number of published papers, along with the country of origin

University	Number of published papers	Country of origin
Damascus University	52	Syria
King Abdulaziz University	2	KSA
European University College	2	UAE
Jouf University	2	KSA
Erciyes University	1	Turkey
King Saud University	1	KSA
Istanbul University	1	Turkey
Kermanshah University of Medical Sciences	1	Iran
Gulf Medical University	1	UAE
Selçuk University	1	Turkey
King Khalid University	1	KSA

Analysis of Keywords

**Figure 5:**
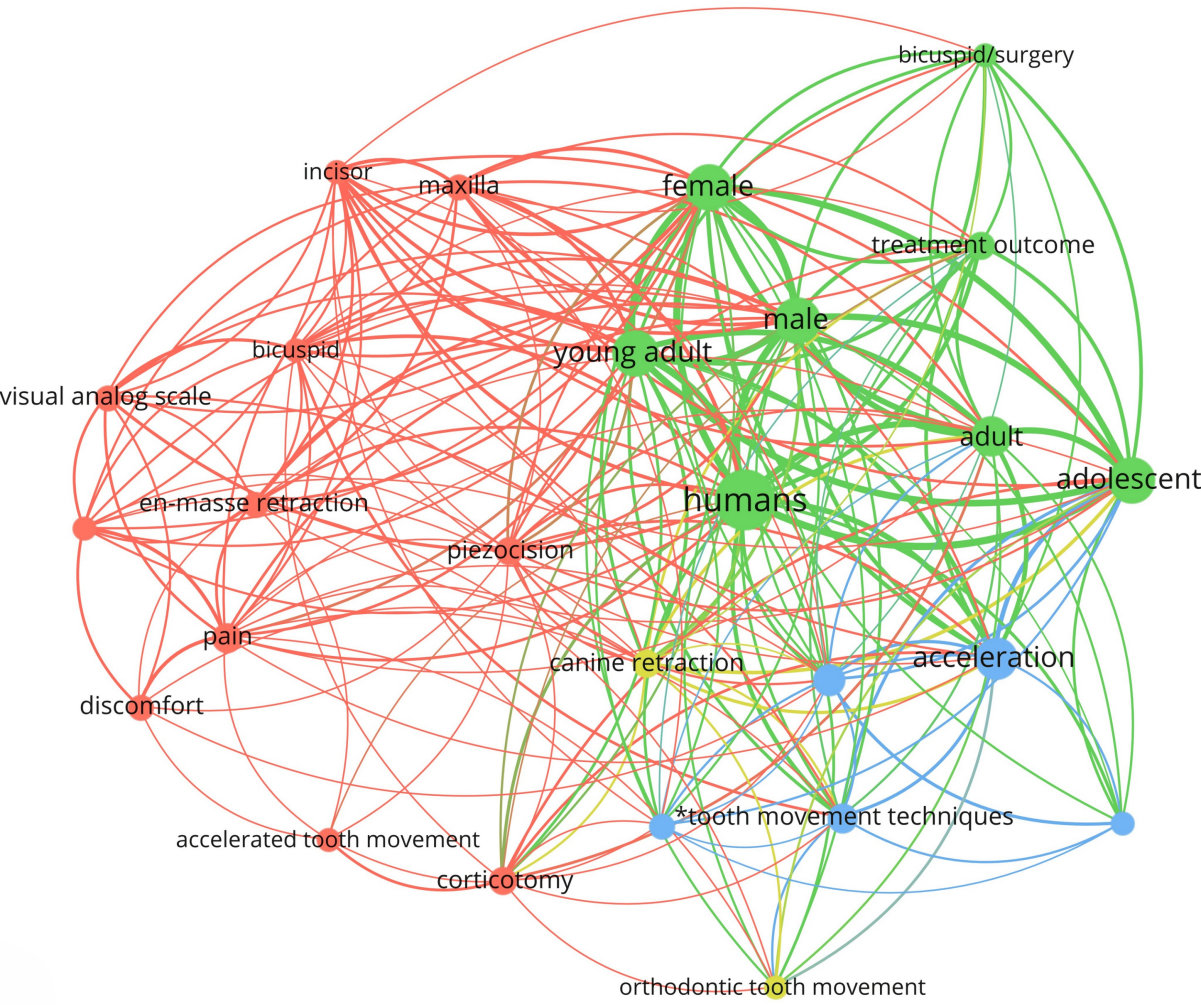
Visual network of the most relevant keywords This figure was created using VOSviewer software (version 1.6.20; Centre for Science and Technology Studies, Leiden University, Leiden, Netherlands).

These keywords were grouped into four clusters, representing distinct research themes. The red cluster focuses on pain and discomfort, with keywords such as pain, pain measurement, tooth extraction, discomfort, and satisfaction. The blue cluster represents various methods for accelerating OTM, including keywords such as piezocision, flapless corticotomy, self-ligating brackets, and LLLT. The green cluster examines the population that requires accelerated OTM, with major keywords including human, adult, adolescent, and OTM. And the yellow cluster focuses on patients’ gender and treatment outcomes, using keywords such as male, female, and young adults (Figure 5).

Discussion

This bibliometric analysis examined 65 publications in accelerated orthodontics from 17 Middle Eastern countries and regions between 2008 and 2025. Key metrics, including publication volume, annual citation frequency, and contributions by author, country, and institution, were assessed. Findings indicate a clear upward trend in both output and citations, reflecting a growing regional interest in accelerated orthodontics research.

The dental research landscape across the Middle East reveals distinct hierarchies that vary by metric and subfield. According to the SCImago Journal & Country Rank (SJR) database [28] in the field of dentistry from the Middle East region (24), Iran and Turkey lead in overall dental publication volume, while Saudi Arabia excels in citation impact, which is attributed to strong international collaborations. Iran also ranks highest in the Middle East for dental publications over the last five years, with 98, followed by Turkey with 95 and Saudi Arabia with 74.

Within this broader context, Syrian institutions demonstrate an unexpectedly high and influential level of productivity specifically in accelerated orthodontics, contributing approximately 80% of the analyzed publications in this field in the Middle East between the periods of 2008 and 2025 (despite significant challenges including political instability, limited resources, and a marked decline in general research output following the 2011 conflict) [29]. This disproportionate output suggests a concentrated and dedicated scholarly effort within Syria’s academic community. Further analysis confirms Syria’s pioneering leadership in accelerated orthodontics, characterized by dual dominance in publication volume and scholarly impact. Notably, Damascus University serves as the central hub of this activity, producing 52 publications, which is more than the combined output of other major regional contributors.

Moreover, Syria’s cumulative citation count of 1,772 far surpasses those of regional peers such as Turkey (170) and Saudi Arabia (115), underscoring the quality and international influence of its research. The remarkable scholarly output is underpinned by the robust institutional foundation in dental and orthodontic education at Damascus University. According to Wang et al., Damascus University was the foremost producer of orthodontic RCTs from 2020 to 2025, a position significantly driven by the high publication activity of a leading researcher (Hajeer MY) who was also cited as the top-publishing author in the field of orthodontics with 23 published RCTs indexed in the Scopus database [30] in the same period of time. Focused research in this field appears to have provided a vital channel for professional contribution and global scholarly engagement. This finding underscores Syria's leading role in the region, indicating it is the most prolific Middle Eastern country in accelerated orthodontics research, as supported by a bibliometric analysis done by de Miranda Ladewig et al., who identified Syria as a top-ranking nation in systematic review output globally [31]. In addition, Syria has the strongest collaboration with other countries and regions, particularly Saudi Arabia. These cooperative efforts across regions in the Middle East can drive further advancements in accelerated OTM.

The analysis of authorship in accelerated orthodontics demonstrates a distinct concentration, both geographically and intellectually, with Syrian-affiliated scholars dominating research output in the field. This Syrian preponderance among the top authors, 14 of the top 15, transcends mere numerical dominance, signifying the country's role as the primary intellectual engine driving scholarly output in this specialty on both regional and global scales. This influential Syrian consortium is responsible for a majority of high-impact publications, fundamentally shaping the global scholarly discourse in accelerated orthodontics. Its influence extends beyond the Middle East, establishing an important position among worldwide orthodontic publication centers [31].

Analysis of the top 10 most-cited articles from 2008 to 2025 reveals the primary research trends. Five articles focused on surgical approaches (e.g., corticotomy, osteoperforation, and piezocision), and five articles focused on LLLT. Highlighting the frequently used methods in accelerating OTM in Middle East countries. This is related to the high demand for orthodontic treatment among a significant adult population in the Middle East, a key driver of the adoption of accelerated methods [32]. This patient demographic is particularly receptive to minor surgical interventions, a tendency reinforced by a cultural propensity to favor surgical solutions [33]. The successful implementation of these invasive techniques is further enabled by well-established interdisciplinary practice models, which facilitate the necessary close collaboration between orthodontists and periodontists or oral surgeons [34]. From a research perspective, these surgical approaches offer distinct advantages, as their clearly defined procedural variables and readily quantifiable outcomes provide a robust framework for designing and publishing compelling clinical studies, thereby reinforcing their prominence in the regional scholarly literature.

Leading the citation count was the 2008 study by Youssef et al., “The effect of low-level laser therapy during orthodontic movement: a preliminary study.” It is recognized as an early application of LLLT in the field of accelerated orthodontics worldwide, ranking third in the published articles investigating the effects of laser on tooth movements, and its high citation rate confirms its fundamental role in LLLT in orthodontics. The second most-cited article was authored by Al-Naoum et al. in 2014 [7]. The authors tested corticotomy and its effects on accelerating upper canine movement by increasing bone turnover, accompanied by demineralization and remineralization at the site of bone injury. This is now recognized as an effective technique in accelerating OTM. Notably, this article is considered an early investigation of traditional corticotomy with flap elevation for accelerated orthodontics, ranking second in appearance among invasive surgical interventions for accelerating OTM, following the study by Aboul-Ela et al. from Egypt in 2011 [35]. Ranking as the third most-cited was the systematic review and meta-analysis by Alfawal et al. [24], which was the first published systematic review and meta-analysis discussing the impact of minimally invasive surgical-assisted orthodontics on the rate of OTM.

The four keyword clusters, delineated by distinct colors, represent primary research subtopics within the field of orthodontic acceleration: pain and discomfort (red), acceleration methodologies (blue), targeted patient populations (green), and patient gender (yellow).

The application of conventional corticotomy techniques in routine orthodontics has been drawing back due to their invasive nature [36]. These procedures necessitate full-thickness mucoperiosteal flaps, which pose risks including postoperative pain, edema, and alveolar bone and gingival loss [37]. Thus, a strong association was observed between invasive acceleration techniques, such as PAOO and corticotomy, and the “pain and discomfort” cluster. This correlation underscores that these surgical interventions are frequently linked with postoperative pain and patient irritation. Notably, these invasive methods were more strongly associated with male patients. Consequently, recent research has shifted its focus towards less invasive alternatives such as piezocision, micro-osteoperforations (MOPs), and flapless corticotomy, which have emerged as prominent areas of investigation [38]. Notably, “low-level laser” was identified as the most frequently used keyword in 2022. Conversely, minimally invasive methods, such as LLLT, showed a negligible association with the “pain and discomfort” cluster. However, these techniques were more frequently associated with female patients, suggesting greater acceptance within this demographic. Both invasive and minimally invasive acceleration methods were associated with the same patient age group, predominantly adolescents.

## Conclusions

This bibliometric analysis of accelerated orthodontics in the Middle East (2008-2025) demonstrates a clear and sustained rise in scholarly activity and impact, with a marked surge from 2021 to 2024. Syria, led by Damascus University, emerged as the dominant contributor in both publication volume and citation impact, reflecting concentrated expertise and strong author networks. Surgical approaches (corticotomy, PAOO, and piezocision) and LLLT represent the most influential research streams, together accounting for the highest-cited works. Keyword clustering revealed four thematic domains: pain and discomfort, acceleration methods, target populations, and gender, highlighting that invasive techniques are closely associated with postoperative pain, while minimally invasive and noninvasive modalities (e.g., LLLT or MOPs) are gaining traction and greater patient acceptability, especially among females. The field shows robust intra-regional collaboration, notably between Syrian and Saudi institutions, but geographic concentration suggests opportunities to broaden multicenter and international trials. Future research should prioritize standardized outcome measures, comparative effectiveness of minimally invasive techniques, and patient-centered evaluations to translate regional advances into wider clinical practice and evidence-based guidelines.

## Appendices

Appendix A

**Table 3:** Search strategy employed in this bibliometric analysis

Database	Search string
PubMed	((((orthodontics) OR (orthodontic treatment) AND ("photobiomodulation" OR “LLLT” OR “Low level laser” OR "micro osteoperforation" OR “MOPs” OR “CAPs” OR "periodontally accelerated osteogenic orthodontics" OR "corticotomy" OR "piezo*" OR "piezocision" OR "surgical-assisted orthoontics" OR "ultrasound" OR "low intensity pulsed ultrasound" OR "laser-assisted corticotomy" OR "vibrat*" OR "high frequency" OR "electromagnetic field" OR "self-ligating brackets") AND ("treatment time" OR "duration" OR "rapid" OR "tooth movement" OR "accelerat*") AND ('SYR" [AD] OR "Syria" [AD] OR "Syrian Arab Republic" [AD] OR "Lebanon" [AD] OR “LBN” [AD] OR "Turkey" [AD] OR “TUR” [AD] OR "Iran" [AD] OR “IRN” [AD] OR "Iraq" [AD] OR “IRQ” [AD] OR "Israel" [AD] OR “ISR” [AD] OR “Oman" [AD] OR “OMN” [AD] OR "Jordan [AD] OR “JOR” [AD] OR "Kuwait" [AD] OR “KWT” [AD] OR "Saudi Arabia" [AD] OR “KSA” [AD] OR "UAE" [AD] OR "United Arab Emirates" [AD] OR "Qatar" [AD] OR “QAT” [AD] OR "Bahrain“ [AD] OR “BHR” [AD] OR “Yemen” [AD] OR “YEM” [AD])))
Web of Science	TS=((orthodontics OR "orthodontic treatment") AND ("photobiomodulation" OR "LLLT" OR "Low level laser" OR "micro osteoperforation" OR "MOPs" OR "CAPs" OR "periodontally accelerated osteogenic orthodontics" OR "corticotomy" OR "piezo*" OR "piezocision" OR "surgical-assisted orthodontics" OR "ultrasound" OR "low intensity pulsed ultrasound" OR "laser-assisted corticotomy" OR "vibrat*" OR "high frequency" OR "electromagnetic field" OR "self-ligating brackets") AND ("treatment time" OR "duration" OR "rapid" OR "tooth movement" OR "accelerat*")) AND AD=("SYR" OR "Syria" OR "Syrian Arab Republic" OR "Lebanon" OR "LBN" OR "Turkey" OR "TUR" OR "Iran" OR "IRN" OR "Iraq" OR "IRQ" OR "Israel" OR "ISR" OR "Oman" OR "OMN" OR "Jordan" OR "JOR" OR "Kuwait" OR "KWT" OR "Saudi Arabia" OR "KSA" OR "UAE" OR "United Arab Emirates" OR "Qatar" OR "QAT" OR "Bahrain" OR "BHR" OR "Yemen" OR "YEM")
Scopus	TITLE-ABS-KEY((orthodontics OR "orthodontic treatment") AND ("photobiomodulation" OR "LLLT" OR "Low level laser" OR "micro osteoperforation" OR "MOPs" OR "CAPs" OR "periodontally accelerated osteogenic orthodontics" OR "corticotomy" OR "piezo*" OR "piezocision" OR "surgical-assisted orthodontics" OR "ultrasound" OR "low intensity pulsed ultrasound" OR "laser-assisted corticotomy" OR "vibrat*" OR "high frequency" OR "electromagnetic field" OR "self-ligating brackets") AND ("treatment time" OR "duration" OR "rapid" OR "tooth movement" OR "accelerat*")) AND AFFIL("SYR" OR "Syria" OR "Syrian Arab Republic" OR "Lebanon" OR "LBN" OR "Turkey" OR "TUR" OR "Iran" OR "IRN" OR "Iraq" OR "IRQ" OR "Israel" OR "ISR" OR "Oman" OR "OMN" OR "Jordan" OR "JOR" OR "Kuwait" OR "KWT" OR "Saudi Arabia" OR "KSA" OR "UAE" OR "United Arab Emirates" OR "Qatar" OR "QAT" OR "Bahrain" OR "BHR" OR "Yemen" OR "YEM")
